# Neoadjuvant Use of Photodynamic Therapy in Basal Cell and Squamous Cell Carcinomas of the Face

**DOI:** 10.5402/2011/809409

**Published:** 2011-05-31

**Authors:** Goran Jeremic, Corey C. Moore, Michael G. Brandt, Philip C. Doyle

**Affiliations:** Department of Otolaryngology-Head and Neck Surgery, Schulich School of Medicine & Dentistry, The University of Western Ontario, London, ON, Canada N6A 5W9

## Abstract

*Background*. This preliminary study sought to determine the success of photodynamic therapy (PDT) in reducing lesion size in an effort to assess the potential application of this treatment approach in a neoadjuvant role. *Objectives*. To quantify the effects of PDT on lesion area (mm^2^) for basal cell and squamous cell carcinomas of the face. *Results*. Eighteen participants (10 BCC lesions and 8 SCC lesions of the face) were assessed. Four lesions (all from the BCC group) showed a complete response to PDT. Of the remaining 14 lesions, 85.7% (*n* = 12) showed reductions in lesion area, while two lesions showed increase in lesion area. Proportional reductions for the 12 lesions that did not demonstrate complete response or an increase in area following-PDT were found to range from 13.2% to 85.1% (BCC) and 6.7% to 89.7% (SCC). *Conclusions*. PDT as a neoadjuvant treatment may provide a simple, efficient, and viable approach to reducing the area of malignant lesions of the face with the advantage of reduced cosmetic and aesthetic morbidities.

## 1. Introduction

Current trends in clinical dermatology practice suggest an increase in the incidence of skin cancers in the region of the head and neck. This would include lesions of the face, an area of the body where cosmetic changes as a consequence of cancer treatment are paramount. Although elimination of the malignancy is the primary emphasis of treatment, the residual side effects of treatment cannot be discounted. While surgical excision remains the recommended approach to the treatment of skin cancers on the face, continued efforts to minimize the impact of treatment must always be carefully considered. 

Skin cancers of the face not only hold significant potential to call attention to themselves, but also have the problem that once treatment is completed, the residual effects of that treatment may continue to pose cosmetic concerns [1]. It is almost certain that any surgical treatment to the face is likely to leave at least some degree of cosmetic change visible to the patient and others. Thus, efforts that seek to reduce the possibility of large resections may carry with it the potential for reduced cosmetic morbidities. In this circumstance, it is not unreasonable to assume that larger lesions will carry the explicit potential for increased posttreatment cosmetic changes that pose personal and social restrictions. Consequently, treatment approaches that create a realistic opportunity to limit the extent of surgical resection offer a considerable attraction in the management of skin cancers of the face. For these reasons, our group has worked toward the goal of evaluating combined methods for the treatment of facial lesions, thereby not only optimizing surgical treatment by reducing the anticipated area of resection, but also doing so with reduced tissue morbidity. 

In an effort to facilitate the ability to meet these multiple desired objectives, we have undertaken the use of photodynamic therapy (PDT) as a precursor to surgical intervention. The emphasis of this approach was borne in hope that utilizing PDT prior to planned surgery could reduce the aesthetic impact of treatment on this important bodily area. Briefly, PDT is able to exploit the intrinsic cellular haembiosynthetic pathway in concert with the principles of photoillumination. By doing so, PDT permits the potential to selectively target malignant cells of the cutaneous system with the advantage of avoiding or reducing collateral treatment impact on nearby regions. One of the potential advantages of PDT for facial lesions is that should surgery be required, the area of resection may be more circumscribed [2]. PDT is not a new method of treatment, but its use in those with lesions of the face is not extensive [3–6]. Based on information provided in the PDT literature to date, this treatment modality appears to be predictable in response and is well tolerated by those who receive it [7], so the inherent treatment limitations appear to be minimal. This is further enhanced if PDT is considered in a neoadjuvant role pending surgery. Although PDT cannot be used in some instances, most particularly in association with basal cell lesions that are characterized by substantial vertical growth (>2-3 mm thickness) or in association with cutaneous lesions that are heavily pigmented [2–6], its potential utility for well-selected cases of malignant lesions of the face is raised. 

These concerns become even more critical when lesions are large in size, a situation that offers the additional challenges of minimizing the sacrifice of lesion-free tissue and directly seeking to reduce negative aesthetic changes. When these concerns are weighed collectively, one can see that treatment of malignant lesions of the face must balance multiple concerns with the desire of not only effecting treatment success from the standpoint of eliminating cancer, but doing so in a manner that explicitly minimizes the resulting surgical defect. Any effort that can facilitate the potential for smaller surgical excisions holds significant potential to ally additional morbidities that center around posttreatment cosmetic defects. Therefore, the question of how effective PDT is if employed presurgically emerges. That is, could PDT be used as an initial treatment with the specific goal of reducing lesion size so that the potential impact of secondary surgery for histologic cure could be achieved? For this reason, we chose to descriptively assess a group of consecutive patients who presented to our clinic with pathologically confirmed malignant lesions of the face. 

## 2. Methods

This study was conducted following full ethical approval through our institutional review board (University of Western Ontario Health Sciences Research Ethics Board, Review number 16430).

### 2.1. Participants

The patient population reported in this paper included 18 participants with confirmed, biopsy-verified nonmelanoma cutaneous lesions. All potential participants were referred to our center, a tertiary care skin cancer clinic for lesion excision and local reconstruction. Of this group, 10 participants had lesions confirmed pathologically to be basal cell carcinoma (BCC), while 8 demonstrated squamous cell carcinoma (SCC), and all were recruited sequentially within our center. Potential participants were excluded if they presented with any lesion directly on the scalp, or one that encroached on the scalp, or lesions on the neck; thus, the participants described had lesions solely on the face. Melanoma was an explicit exclusion criterion, as were in situ carcinomas. 

An additional inclusion criterion, is that all participants presented with a malignant lesion on the face (BCC or SCC) judged by one of the authors (C. C. Moore) as not being amenable to surgical excision due to its size (in mm^2^), having the anticipated extent of necessary resection with appropriate oncologic margins, and/or the anatomical proximity of the lesion and the anticipated surgical excision to remove the lesion from aesthetically sensitive areas (e.g., eyes, nose, and lips). Because of the high potential for cosmetic and aesthetic morbidity associated with these lesions due to size and location on the face, they were deemed to be ideal candidates for participation. Finally, participants were excluded from consideration if they were unable to self-administer the photosensitizing agent or if they exhibited possible sensitivity for PDT (i.e., history of cutaneous photosensitization, porphyria, hypersensitivity to porphyrins, and/or photodermatosis) [8–10]. 

### 2.2. Procedure

Following a full evaluation by one of the authors (CCM), each of the 18 participants was instructed by a clinical nurse in how to apply a topical photosensitizer (5 mg of 10% concentrated 5-aminolevulinic acid powder mixed with an emulsifying Glaxal Base gel) to the lesion. After individual instruction and confirmation that the application process was fully understood and questions answered, each participant was then asked to return on a predetermined date. Participants were asked to self-apply the photosensitizing agent three hours prior to their scheduled appointment. The application of the photosensitizer 3 hours prior to the scheduled appointment maximizes its absorption [11–14].

#### 2.2.1. Measurement of Lesion Area

Upon arrival at the skin clinic, the residual photosensitizer was first wiped from the lesion, and the lesion was fluoresced, demarcated, and recorded [15]. Fluorescence was completed using a filtered lamp that emitted a spectra of photoenergy in wavelengths that ranged from 320 to 400 nm. Under fluorescence, the entire border of the lesion was identified directly on the participant's skin by using a surgical marking pen for demarcation. Once demarcation was completed, a clear acetate film was placed over the entire area of the lesion, and its full border was carefully transcribed. Dimensions of interest included the full length and width of the lesion circumscribed under photodynamic photodelineation. Lesion length was the diameter of the greatest magnitude measured at a precision of 0.5 mm. This procedure provided an index of lesion size in mm^2^ that was identified as the baseline or pretreatment (PDT) measure. All measures were obtained with reference to the inner marking of the lesion border; this process served to reduce potential measurement artifact associated with the width of the surgical marker. Subsequently, the entire area of the lesion was calculated using the formula (length/2) × (width/2) × pi. Measures of post-PDT lesion were taken again using the identical acetate film demarcation procedure described for the pre-PDT measure. However, post-PDT measures were generated independently by two of the authors (C. C. Moore and G. Jeremic).

Following this process, the participant then received the standard PDT. Briefly, this protocol involved a sequence of two 20-minute light exposures; the light exposure was completed using a diode lamp that emits light in wavelengths of 633 nm ± 10 nm range. The two treatment exposures during this single PDT session were completed with a one-hour dark interval between exposures. Following the initial PDT treatment, all participants returned to the clinic and their lesions were measured again using an identical procedure to that described for the baseline, pretreatment measure. This second measure obtained at 4 weeks was identified as the posttreatment measure.

### 2.3. Descriptive Analysis of Data

Data related to lesion area for all the 18 participants was assessed on an individual participant basis; however, evaluation of pre- and post-PDT assessments was considered according to histologic grouping (BCC and SCC) based on unique history of both types of lesions (Table 1). Because this project was descriptive in nature with the objective focused on assessing whether PDT reduced lesion size, and more specifically, the proportional extent of that reduction, parametric statistics were not used. Again, the primary goal of our assessment sought to determine if PDT resulted in changes in lesion area (in mm^2^) following a single session of PDT and the associated absolute and proportional level of reduction if observed following PDT. 

## 3. Results

### 3.1. Basal Cell Carcinoma

**Figure 2:**
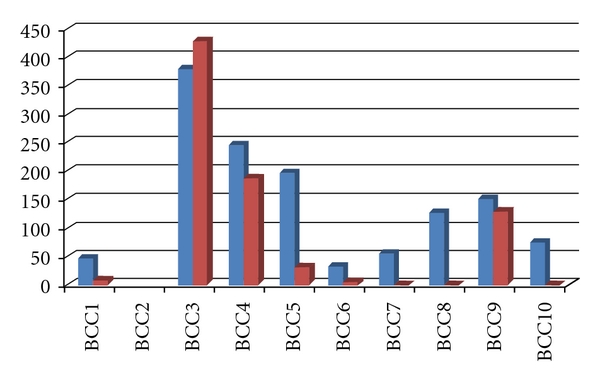
Pre-PDT (blue) and post-PDT (red) measures of lesion area (in mm^2^) for participants with BCC lesions (*n* = 10). Participant BCC2 has been removed because his pre-PDT lesion was >1700 mm^2^, thus, altering the scale of change from the remaining 9 participants in this group; however, this participant did have a complete response to PDT.

Pre-PDT lesion area measures for participants in the BCC group ranged from 31.4 mm^2^ to 1727.8 mm^2^ with a mean of 303.3 ± 120.93 mm^2^. Of the 10 lesions in this group of participants, 4 were found to respond completely to treatment; upon reassessment at 4 weeks following -PDT under fluorescence, there was no visible lesion observed. In contrast to these four participants, one participant exhibited an increase in the lesion area from 377 mm^2^ before-PDT to 428.8 after-PDT measurement. For the remaining five participants with BCC, the post-PDT measures revealed lesion areas that ranged from 4.7 mm^2^ to 188.5 mm^2^. For those who did not have a complete response to PDT, a proportional reduction from pre-PDT lesion area was found to range from 13.2% to 85.1% from baseline lesion area measures (Figure 2). Visual inspection of the raw data indicated no apparent trend in the extent of lesion area decrease based on initial measures (Table 2). Interestingly, however, the largest BCC lesion (area = 1727.82 mm^2^) showed a complete response while the smallest (area = 31.4 mm^2^) showed a very good, yet incomplete, response (post-PDT measure = 4.7 mm^2^, proportional reduction = 85.1%). 

### 3.2. Squamous Cell Carcinoma

**Figure 1:**
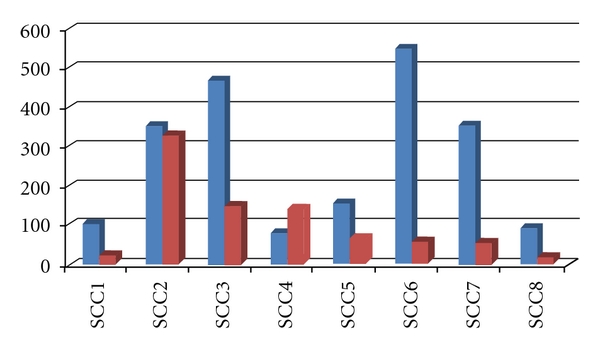
Pre-PDT (blue) and post-PDT (red) measures of lesion area (in mm^2^) for participants with SCC lesions (*n* = 8).

Pre-PDT area measures for the SCC lesions ranged in size from 80.1 mm^2^ to 552.1 mm^2^ (mean area = 270.7 mm^2^). Post-PDT area measures ranged from 19.6 mm^2^to 329.9 mm^2^ (mean area = 106.8 mm^2^). Unlike some of those in the BCC group, of the 8 participants in this group, none were found to respond completely to this single PDT treatment; similar to that observed on the BCC group, one SCC lesion was found to increase following PDT (from 80.1 mm^2^ to 141.4 mm^2^) (Figure 1). Yet for the remaining 7 lesions, when pre- and post-PDT measures were assessed, we found a proportional reduction in lesion area from baseline measures that ranged from 6.7% to 89.4%. As with the BCC lesion group, no discernable trend was identified in the extent of lesion area reduction following PDT (Table 1). 

## 4. Discussion

The purpose of this descriptive project is to assess the potential value of topical PDT as an initial treatment for skin cancers of the face. The rationale for seeking this information was contingent upon the desire to reduce lesion size so that the potential impact of secondary surgery for histologic cure might be achieved. For this reason, we chose to descriptively assess a group of consecutive patients who presented to our clinic with pathologically confirmed malignant lesions of the face with the goal of quantifying the area of their lesions pre- and post-PDT. In seeking to determine the viability of using PDT as a treatment modality for skin cancers of the face, we also included a sequential series of patients who presented to our center and who represented a wide range of lesions sizes; this included an evaluation of 18 participants, 10 who presented with BCC lesions and 8 with SCC lesions. If the area of a given lesion could be reduced via PDT, it was our belief that definitive treatment in the form of surgical excision could potentially be less aggressive from the standpoint of tissue excision [15–17]. Consequently, the potential cosmetic and aesthetic impact of treatment may be reduced with improved patient outcomes without sacrificing oncologic principles of treatment. 

Data gathered from this preliminary study suggest that PDT may serve as a valuable treatment modality for malignant lesions of the face. Based on the data obtained, it appears that PDT can be of value for both BCC and SCC facial lesions. While two of the 18 participants included in the study, one who presented with a BCC and the other with a SCC, were found to have increased lesion areas at the time of post-PDT measures, 16 (89%) demonstrated reductions in lesion area following a single PDT treatment. This finding supports the potential application of PDT in a neoadjuvant role for facial lesions where the concern of cosmetic and aesthetic morbidity is not insignificant [18–21]. Additionally, our data revealed that 4 (25%) of the 16 participants who demonstrated a positive response to PDT (i.e., a reduction in lesion area) showed a complete response. All these four cases came from the BCC group which may indicate a particular histological sensitivity to PDT. Although this assumption cannot be generalized at present, these preliminary data provide initial validation of the utility of PDT as a neoadjuvant therapy for BCC lesions of the face. However, further study is clearly required in order to further substantiate and validate this interpretation.

The desire to reduce the potential negative impact of surgical resections of malignant lesions of the face carries considerable value in the context of the patients satisfaction as a treatment outcome. If surgical intervention can be performed without loss of oncologic safety, but at the same time strive to reduce the size of resection, the personal impact on the patient is likely to be substantial. Facial lesions and the aesthetic consequences of the treatment of such lesions carry a social impact that hold potential for great disability. Although some degree of residual cosmetic defect is almost always going to be present in treatment of any facial lesion, if the defect can be reduced to a minimum, multiple outcomes may be enhanced. As a result, we believe that PDT may serve a valuable role as neoadjuvant therapy for malignant lesions of the face. It is clear that surgery remains the gold standard specific to treatment of skin cancers of the face, yet the ability to minimize the posttreatment consequences of surgery cannot be discounted.

In respect to the present work, several limitations must be noted. First, we have documented the present data solely in a descriptive fashion. No comparison was undertaken in an effort to identify whether the reduction in lesion area was “statistically significant.” Our reason for not performing such analyses was twofold. First, the sample population required in order to provide a justifiable confirmation of the effectiveness would have to have been substantial. Given that the lesion size is likely to be highly variable from person to person both prior to and following treatment, the ability to distinguish a significant change is fraught with problems. In this vein, the more critical determination centers on whether or not a given lesion can be reduced in size and what extent of proportional reduction in size can be confirmed. As observed in the present work, 16 of 18 participants were documented to have reduction in lesion area following PDT with four exhibiting a complete response. Thus, the second and perhaps a more important reason for documenting the absolute and subsequent proportional change in lesion area as a more valuable index was predicated on the notion that it was always anticipated that surgery would be performed. PDT was used in an effort to reduce lesion area so that surgery could be optimized with the least likelihood of facial disfigurement. Therefore, if a lesion area could be reduced, surgical excision could be less extensive with subsequent cosmetic value without oncologic risk. It is our impression that the present data provide valuable information indicating that PDT should be considered as an adjuvant treatment modality for malignancies of the face. While further research is required, the present findings on PDT appear to provide support for continued use and exploration in the context of treatment approaches for BCC and SCC lesions of the face. Consequently, we believe the PDT offers a relatively simple and cost-effective means as a neoadjuvant modality for facial lesions with associated advantages in surgical efforts to reduce the cosmetic and aesthetic impact of surgical intervention.

## Figures and Tables

**Table 1:** Participant demographics.

	BCC	SCC
Mean age	81	78
Gender		
Female	6	2
Male	4	6

Total number of lesions	10	8

**Table 2:** Pre-PDT and post-PDT lesion size (in mm^2^) and the proportional change in lesion area size (listed as a percentage of change from baseline) for BCC and SCC participant groups.

BCC group			SCC group		
Pre-PDT	Post-PDT	Proportional change	Pre-PDT	Post-PDT	Proportional change
47.1	9.4	20.1	106.0	28.3	73.4
1727.8	0.0	100	353.4	329.9	6.7
377.0	428.8		471.2	148.4	67.7
245.5	188.5	23.1	80.1	141.4	
197.9	31.4	84.4	155.5	66.8	57.1
31.4	4.7	85.1	552.1	58.9	89.4
55.0	0.0	100	353.4	55.0	84.5
127.2	0.0	100	94.2	19.6	79.2
149.2	129.6	13.2			
75.4	0.0	100			
